# The association of workplace smoke-free policies on individual smoking and quitting-related behaviours

**DOI:** 10.1186/s12889-021-12395-z

**Published:** 2021-12-20

**Authors:** Haoxiang Lin, Min Li, Meijun Chen, Yihua Liu, Yuting Lin, Zhao Liu, Zhijie Zheng, Chun Chang

**Affiliations:** 1 School of Public Health, Peking University Health Science Center, Beijing, China; 2 DeZhou Center for Disease Control and Prevention, Dezhou, China; 3 Tobacco Medicine and Tobacco Cessation Center, China-Japan Friendship Hospital, Beijing, China

## Abstract

**Background:**

Our study aims to provide information about workplace smoke-free (SF) policy coverage in mainland China and to assess the relationship between workplace SF policies and secondhand smoke (SHS) exposure, current smoking, smoking harm awareness and quitting intention among smokers.

**Method:**

Data from the 2018 Asia Best Workplace Mainland China programme were used to address these aims. This cross-sectional study included 14,195 employees from the 2018 survey and 14,953 employees from the 2019 survey. Logistic regression with year-fixed effects was applied to these data. The dependent variables were SHS exposure, smoking or smoking harm awareness. The explanatory variable was the SF workplace policy.

**Results:**

A total of 21,275 participants (73.0%) reported working under SF policies. The overall prevalence of smoking and SHS exposure were 20.3% and 52.5%, respectively. The workplace SF policy was significantly associated with lower SHS exposure (OR: 0.48, 95% CI: 0.45–0.51), lower current smoking employees (OR: 0.81, 95% CI: 0.76–0.87) and higher awareness of smoking harm (OR: 1.76, 95% CI: 1.61–1.91). However, workplace SF policy was not significantly associated with quitting intention (OR: 0.99, 95% CI: 0.84–1.16).

**Conclusion:**

Our study identified that although most companies had established workplace SF policies, the overall prevalence of SHS exposure remained very high. Workplace SF policy is associated with lower SHS exposure, lower overall current smoking and higher awareness of smoking harm. These findings provide valuable evidence to promote such policies in all workplaces.

**Supplementary Information:**

The online version contains supplementary material available at 10.1186/s12889-021-12395-z.

## Background

Second-hand smoke (SHS) contains hundreds of toxic and carcinogenic chemicals, and involuntary SHS exposure contributes to premature death and causes various diseases in nonsmokers [1, 2]. The workplace is a major source of SHS exposure for adults, and the negative influence incurred by SHS exposure affects workplace productivity and contributes to absenteeism [3].

According to previous studies, the smoke-free (SF) policy is a key contributor to the decline in SHS exposure. As of July 2019, 25 US states had adopted comprehensive 100% SF laws in workplaces, restaurants and bars, which caused a sharp decrease in SHS exposure from 52.5% during 1999–2000 to 25.2% during 2013–2014 [4]. Since China ratified the Framework Convention on Tobacco Control (FCTC) [5] in 2005, considerable progress has been made to reduce SHS exposure in China, particularly by implementing SF policies. As a result, according to the Chinese Adult Tobacco Survey, the prevalence of SHS exposure was 63.3% in 2010 [6], 54.3% in 2015 [7], and 50.9% in 2018[8].

Despite the processes, China currently does not have national legislation for workplace SF policy [9], and the implementation of workplace SF policies and their impact have not been fully investigated. Most studies that examine the association between workplace SF policy and SHS exposure have been conducted in Western countries [10, 11]. For example, a systematic review in the British Medical Journal showed that smoke­free workplaces are associated with reductions in the prevalence of smoking of 3.8% and 3.1 fewer cigarettes smoked per day per continuing smoker [12]. Kahraman et al. found that working in SF workplaces was associated with increased rates of quitting smoking and with increased use of Maras powder, which is a local form of oral smokeless tobacco in Turkey [13].

In China, one study in 2010 reported that workplace SF policy had significant associations with lower smoking prevalence and daily cigarette consumption but not with employee quitting intentions; however, this study only collected 1070 questionnaires from one company in Shanghai [14]. Until recently, two Chinese studies reported the association of workplace SF policies and possible effects and showed that SF workplace policies are associated with lower SHS exposure and broader spillover effects, such as reduced drinking behaviour [15, 16]; however, both studies used only one-year cross-sectional data. To the best of our knowledge, no other recent studies have been conducted on this topic. Such knowledge is critically needed to help inform policymakers and health practitioners to establish stronger SF policies and conduct campaigns and interventions to reduce exposure to workplace SHS in China.

To help fill the evidence gap, we report the findings from a cross-sectional survey to provide valuable evidence to promote such policies. Specifically, we aim to (1) provide information about the coverage of workplace SF policies in mainland China and (2) assess the relationship between workplace SF policies and SHS exposure, current smoking, smoking harm awareness (SHA) and quitting intentions (QI) among smokers.

In addition, a previous study found sex differences in SHS exposure and knowledge of the health hazards of smoking in 12 provinces in China [17]. Given the huge difference in smoking prevalence in China (50.5% of adult males and 2.1% of adult females are smokers) [8], we think that it is helpful to report sex differences in SF workplaces on smoking behaviour.

## Methods

### Settings

The Asia Best Workplace Mainland China (ABWMC) programme was a cross-sectional survey to support companies in building healthy workplaces through policy, infrastructure, and culture. The ABWMC programme was designed by Peking University and organized by the American International Assurance Company. We invited companies to join the programme using a purposive selection method. The inclusion criteria for participating companies were as follows: (1) legal companies registered in China; (2) at least 100 full-time employees; and (3) agreement to participate in the programme.

### Population

We used the 2018 and 2019 ABWMC programme data. The total sample size was 29,148, of which there were 14,195 from the 2018 survey and 14,953 from the 2019 survey.

### Sampling

The human resource departments of each company delivered the questionnaires to all employees. All employees who were (1) aged 18 years old or above and (2) full-time employees were invited to participate in this programme.

### Data collection procedure

A four-stage method was applied in this survey. In the first stage, experts at Peking University designed standardized questionnaires, including sociodemographic information, smoking-related behaviour and quitting intentions (Additional file 1: Research Questionnaire). In the second stage, an online survey system was developed by Ipsos Inc., and a specific internet link was generated. The self-check function of the online survey system automatically identified missing data, logical errors and illegal characters. In the third stage, the Human Resources Departments of each participating company delivered the internet link to all employees. After signing the informed consent online, the employees completed and submitted the questionnaire. All participants knew that the statistical analyses would be anonymously conducted, and their information would be used for research purposes. At the final stage, the submitted questionnaires were reviewed by the staff at Peking University, and the respondents were contacted for clarifications if any problems were detected. This study was approved by the Peking University Institutional Review Board (number: IRB00001052-18,055).

### Measurements

#### SHS exposure and workplace SF policies

The presence of SHS exposure was measured using the question “How many days per week do you usually suffer from SHS exposure at the workplace for more than 15 min a day? A: almost every day; B: 4–6 days; C: 1–3 days; D: never”. Respondents who answered D were considered to be working without SHS exposure. The presence of a workplace SF policy was measured using the question “Does your company have SF policies? A: no SF policies; B: SF policies that permit smoking in parts of the indoor area; C: SF policies that completely ban smoking inside the building; D: I have no idea”. Respondents who answered C were considered to be working under an SF policy.

#### Smoking and quitting intentions

Smoking was measured by the following question: “Do you smoke now? A: yes, every day, B: yes, only occasionally, C: I have quit smoking, D: never”. Respondents who answered A or B were considered current smokers. Quitting intentions were measured using the question “Are you going to quit smoking? A: yes, within a month, B: yes, within 6 months, C: yes, but not within 6 months, D: no plan for quitting”. Respondents who answered A, B or C were considered intending to quit smoking.

#### Smoking harm awareness

Smoking harm awareness was measured by the following question: “To the best of your knowledge, which diseases can be caused by smoking? A: stroke, B: heart disease, C: lung cancer, D: cardiovascular disease, E: chronic obstructive pulmonary disease, F: asthma, G: I don’t know.” Respondents who chose all answers from A to F were considered to have smoking harm awareness.

#### Other control variables

In addition to these key variables, other variables were collected as control variables, such as sex, age, marital status, ethnicity, education, chronic diseases, job position and night-shift duty.

#### Data analysis

Our data have a hierarchical structure; therefore, we first tried to use hierarchical linear modelling by setting individual- and company-level factors. This type of analysis will consider that workers' responses are correlated within companies. We ran four standardized models (null model, random coefficients regression model, intercepts as a model, slopes as an outcomes model). However, when we finished the null model, we found that the intraclass correlation coefficient (ICC) was too low (0.051, lower than 0.059), which indicates that only approximately 5.1% of the total variation was attributable to differences among companies/clusters [18]. In other words, we can use the usual method to perform analyses. Therefore, we used logistic regression for our statistics.

Logistic regression was used to estimate the association of workplace SF policies with individual smoking and quitting behaviours. The specification of our empirical model was as follows:

$$\mathrm{SHS exposure }\left(\mathrm{or smoking}\right)= {\upbeta }_{0+}{\upbeta }_{1} \mathrm{SF policy}+\upgamma {\mathrm{X}}_{\mathrm{i}}+{\upgamma }_{t}+{\varepsilon }_{it}.$$

The dependent variable was either SHS exposure or smoking. The explanatory variable SF policy was a dummy that indicates whether an employee was working for a company with an SF workplace policy (if yes = 1; otherwise = 0). $\gamma Xi$ is a variety of other control variables; ${\gamma }_{t}$ is the year-fixed effects to control for year-specific factors, and ${\varepsilon }_{it}$ is the error term. We also separately performed regression with the sample of each year.

To determine whether such a policy had an association with smoking harm awareness and quitting intention, we changed the dependent variable and ran the following regression:

$$\mathrm{SHA }\left(\mathrm{or QI}\right)={\upbeta }_{0+}{\upbeta }_{1} \mathrm{SF policy}+\upgamma {\mathrm{X}}_{\mathrm{i}}+{\gamma }_{t}+{\varepsilon }_{it.}$$

Our analyses used the responses to the questionnaire for whom the variables of interest were available, with no imputation for missing data. All statistical analyses were performed using SPSS 19.0.

#### Role of the funding source

The funders of the study had no role in the design of the study, collection, analysis, or interpretation of the data, or the writing of the paper.

## Results

In total, 79 companies participated in the 2018 ABWMC programme. Of them, 53.2% were private Chinese companies, 32.9% were foreign companies, 7.6% were state-owned companies and 6.3% were joint ventures; 39.2% of the companies were located in Shanghai, 26.6% in Guangdong Province, 20.3% in Beijing, 11.4% in Jiangsu Province and 2.5% in Sichuan Province; 84.8% of the companies implemented a workplace SF policy.

In 2019, 85 companies participated in the programme. Of them, 43.5% were foreign companies, 42.4% were private Chinese companies, 11.8% were joint ventures, and 2.4% were state-owned companies; 52.9% were located in Shanghai, 14.1% in Jiangsu Province, 11.8% in Guangdong Province, 10.6% in the city of Beijing, 3.5% in the city of Shenzhen, and 7.1% in other cities; 94.1% of the companies implemented a workplace SF policy.

In all companies, 29,148 individuals participated in this study and submitted their responses to the self-administered questionnaires. The characteristics of the participants are shown in Table 1. A total of 46.7% of the study population was male, with an average age of 31.78 ± 7.11 years. The majority of participants were married (59.9%) and held bachelor’s degrees or above (86.1%); 21,275 participants (73.0%) reported working under SF policies. The overall prevalence of smoking and SHS exposure was 20.3% and 52.5%, respectively; of the total SHS exposure, the proportion of workers with SHS exposure every day was 15%. Unfortunately, only 15.0% of participants had comprehensive awareness of smoking harms.

**Table 1:** Demographics and basic characteristics of the study population

	**Full (n/%)**	**2018 (n/%)**	**2019 (n/%)**
**Age**			
16–29	12,179(41.8)	6228(43.9)	5951(39.8)
30–39	12,852(44.1)	5932(41.8)	6920(46.3)
40–49	3643(12.5)	1794(12.6)	1849(12.4)
50 and above	474(1.6)	241(1.7)	233(1.6)
Mean age (SD)	31.78 ± 7.112	31.60 ± 7.273	31.95 ± 6.951
**Sex**			
Male	13,622(46.7)	6408(45.1)	7214(48.2)
Female	15,526(53.3)	7787(54.9)	7739(51.8)
**Ethnicity**			
Han	27,888(95.7)	13,594(95.8)	14,294(95.6)
Others	1260(4.3)	601(4.2)	659(4.4)
**Marriage**			
Single	11,015(37.8)	5534(39.0)	5481(36.7)
Married	17,448(59.9)	8491(59.8)	8957(59.9)
Divorced or widowed	329(1.1)	170(1.2)	159(1.1)
**Education attainment**			
Middle school or lower	761(2.6)	476(3.4)	285(1.9)
High school	3301(11.3)	2080(14.7)	1221(8.2)
College	22,033(75.6)	10,374(73.1)	11,659(78.0)
Master and above	3053(10.5)	1265(8.9)	1788(12.0)
**Smoking**			
Yes	5912(20.3)	2955(20.8)	2957(19.8)
No	23,236(79.7)	11,240(79.2)	11,996(80.2)
**Workplace SF policy**			
Yes	21,275(73.0)	10,178(71.7)	11,097(74.2)
No	7873(27.0)	4017(28.3)	3856(25.8)
**Secondhand smoke exposure**			
Never	13,858(47.5)	6429(45.3)	7429(49.7)
1 to 3 days	8906(30.6)	4423(31.2)	4483(30.0)
4 to 6 days per week	1931(6.6)	981(6.9)	950(6.4)
Every day	4453(15.3)	2362(16.6)	2091(14.0)
**Smoking harm awareness**			
Yes	4375(15.0)	1779(12.5)	2596(17.4)
No	24,772(85.0)	12,416(87.5)	12,356(82.6)
**Job position**			
Not administrative	19,449(66.7)	9458(66.6)	9991(66.8)
Administrative	9699(33.3)	4737(33.4)	4962(33.2)

As shown in Table 2, after adjusting for age, education and marriage and controlling for year-fixed effects, the workplace SF policy was significantly associated with lower SHS exposure in the full model (*OR*: 0.48, 95% CI: 0.45–0.51). The pattern was similar for all other subgroup analyses.

**Table 2:** Adjusted odds ratio (*OR*) and 95% confidence intervals for the SHS exposure, current smoking, smoking awareness and quitting intention

	**SHS exposure**	**current smoking**	**smoking harm awareness**	**quitting intention**
**Male**				
**SF workplace policy**	0.44**(0.39–0.48)	0.83**(0.76–0.90)	1.77**(1.57–2.01)	1.04(0.88–1.23)
**Education**				
Master and above	Ref	Ref	Ref	Ref
Bachelor degree	1.51**(1.30–1.74)	1.76**(1.54–2.01)	0.89 (0.77–1.03)	0.86 (0.60–1.25)
High school	2.01**(1.63–2.47)	3.67**(3.13–4.35)	0.52**(0.42–0.65)	0.69 (0.471.03)
Middle school and lower	1.72**(1.23–2.41)	3.13**(2.46–4.00)	0.34**(0.22–0.53)	0.85 (0.51–1.42)
**Chronic disease**				
No	Ref	Ref	Ref	Ref
Yes	1.31**(1.17–1.46)	1.06 (0.98–1.16)	0.96 (0.85–1.07)	1.09 (0.91–1.29)
**Year effect**				
2018	Ref	Ref	Ref	Ref
2019	0.93 (0.85–1.03)	0.93 (0.86–1.00)	1.39** (1.26–1.54)	1.19 *(1.01–1.39)
**Female**				
**SF workplace policy**	0.51**(0.47–0.55)	0.96(0.80–1.16)	1.74**(1.54–1.96)	0.42*(0.19–0.95)
**Education**				
Master and above	Ref	Ref	Ref	Ref
Bachelor degree	1.80**(1.60–2.03)	1.36 (0.98–1.85)	1.07 (0.92–1.24)	0.80 (0.18–3.51)
High school	2.23**(1.88–2.63)	1.84**(1.23–2.76)	0.68**(0.53–0.86)	0.65 (0.13–3.25)
Middle school and lower	1.51**(1.16–1.95)	1.63 (0.90–2.96)	0.29**(0.17–0.50)	1.75 (0.16–18.70)
**Chronic disease**				
No	Ref	Ref	Ref	Ref
Yes	1.35**(1.24–1.47)	1.38**(1.15–1.66)	0.90 (0.80–1.00)	1.09 (0.53–2.26)
**Year effect**				
2018	Ref	Ref	Ref	Ref
2019	0.88**(0.82–0.94)	0.80* (0.67–0.96)	1.41** (1.29–1.55)	1.00 (0.52–1.95)
**All sample**				
**SF workplace policy**	0.48**(0.45–0.51)	0.81**(0.76–0.87)	1.76**(1.61–1.91)	0.99(0.84–1.16)
**Education**				
Master and above	Ref	Ref	Ref	Ref
Bachelor degree	1.68**(1.53–1.84)	1.53**(1.36–1.72)	0.98 (0.88–1.08)	0.87 (0.61–1.24)
High school	2.11**(1.86–2.41)	2.88**(2.50–3.31)	0.60**(0.51–0.70)	0.69 (0.47–1.01)
Middle school and lower	1.56**(1.28–1.92)	2.55**(2.09–3.11)	0.32**(0.23–0.45)	0.89 (0.54–1.46)
**Chronic disease**				
No	Ref	Ref	Ref	Ref
Yes	1.34** (1.25–1.43)	1.19** (1.11–1.28)	0.93 (0.86–1.00)	1.10 (0.93–1.30)
**Year effect**				
2018	Ref	Ref	Ref	Ref
2019	0.90**(0.85–0.95)	1.05 (0.98–1.11)	1.40** (1.30–1.50)	1.17* (1.00–1.37)

Note: The model was adjusted for age, education attainment, ethnicity, marriage, chronic disease, position and night-shift duty. In this table, where a sample was composed of men and women, sex was added as another independent variable

From the Second Column of Table 2, the SF policy was significantly associated with fewer smokers in the full sample (*OR*: 0.81, 95% CI: 0.76–0.87) and male sample (*OR*: 0.83, 95% CI: 0.76–0.90). In addition, the *OR* values of the female models were not statistically significant.

To examine the association of SF workplace policies with smoking harm awareness, we reconducted the regressions, and the results are presented in the third column of Table 2. SF policy was significantly associated with higher awareness of smoking harm (*OR*: 1.76, 95% CI: 1.61–1.91). The effect was strong and significant for both male and female models.

The last column of Table 2 reports the association between such policy and smokers’ quitting intention. We found that workplace SF policy was not significantly associated with quitting intention in most models.

When two years were compared, 2019 participants reported lower second-hand smoke exposure (*OR*: 0.90, 95% CI: 0.85–0.95), higher smoking harm awareness (*OR*: 1.40, 95% CI: 1.30–1.50) and stronger quitting intention (*OR*: 1.17, 95% CI: 1.00–1.37).

One finding, which is not our main result but is very interesting and deserves some attention, is that the SHS exposure (*OR*: 1.34, 95% CI: 1.25–1.43) and current smoking (*OR*: 1.19, 95% CI: 1.11–1.28) were significantly associated with chronic disease. Future studies can use the longitudinal method to more accurately explore such associations.

## Discussion

To the best of our knowledge, our study is one of the largest surveys to date to evaluate the possible association of workplace SF policies on SHS exposure and smoking-related behaviour in a Chinese population. The results show that: (1) most companies had established workplace SF policies; however, the overall prevalence of SHS exposure in workplaces (52.5%) remains very high. For example, India is a large country with a large population that is always believed to be comparable to China in many aspects, but the overall prevalence of SHS exposure in workplaces is 30.2% [19]. The Russian Federation is also a large country with a high smoking prevalence, but the overall prevalence of SHS exposure in workplaces is 21.9% [20]. (2) Workplace SF policies show a strong association with lower SHS exposure, fewer current smokers and higher awareness of harm from smoking.

Article 8 of the WHO FCTC requires parties to adopt and implement measures to reduce exposure to tobacco smoke in indoor workplaces, indoor public places, public transport and other public places [5]. Since the FCTC was ratified in 2005, the Chinese government has made a serious commitment to reduce or eliminate SHS exposure in workplaces. The prevalence of SHS exposure was reduced from 63.3% in 2010 [6] to 50.9% in 2018 [8], which was similar to the 54.7% found in our study. Moreover, our study found a strong association between workplace SF policies and lower SHS exposure, which is consistent with a systematic review which suggests that SHS exposure in the workplace decreased significantly from 20 to 8% after an SF policy was implemented [21].

As an impressive finding, our data suggest that SF workplace policy is associated with better smoking harm awareness. Our findings reinforce the recommendations of a previous study that SF policies may spread SF norms in the workplace. Furthermore, our study actually supports the possibility of spillover impacts of SF workplace policies (on current smoking and harm awareness of smoking). How do these factors interact with one another? Is there any potential mediator? All of these questions merit further research. A detailed description of the internal mechanisms and possible pathways has been reported elsewhere [22].

In general, workplace SF policies should motivate smokers to quit by making it more difficult to smoke; for example, smokers must go out of their workplace to smoke and increase the time cost of smoking [19]. However, consistent with the findings of a previous study [11], no significant association was found between workplace SF policies and more positive quitting intention. One possible explanation is that the “hardcore” smokers may be less motivated to quit regardless of SF policies at worksites; therefore, more intensive intervention should be provided for them [23]. Another explanation is the violation of workplace SF policy. A workplace SF policy is undermined when it is violated by many smokers who continue to smoke, and this noncompliance may jeopardise the quitting intention of a smoker because he/she is surrounded by others who continue to smoke [24].

We also found a reverse association between SF policies and smoking status. However, we did not find an association with quitting intention, so fewer smokers may not attribute to more positive quitting behaviour. Nonsmokers are more likely to select companies with SF policies. Future research will benefit from exploring other potential factors related to this.

The comparison between sexes did not show a significant difference in SHS exposure and smoking harm awareness, which suggests that a SF policy can generate a positive effect on both males and females. Since female smokers only accounted for 4.1% of the sample, we should focus more on males when explaining the results on quitting intention. The 2019 participants reported lower SHS exposure, higher smoking harm awareness and stronger quitting intention, which may be explained by the participating companies, many of which are based in Shanghai (39.2% in 2018 and 52.9% in 2019). Shanghai is the economic centre of China and has more international communication, willingness and capability to organize tobacco control activities. For example, the Shanghai City Health and Family Planning Commission provided persistent and strong support to develop SF legislation and its enforcement [9]. Therefore, it is believed that with more companies from Shanghai joining the ABWMC programme in 2019, participants may show more positive tobacco control awareness.

Our study has several limitations. First, we only use cross-sectional data for this estimation. As a result, we cannot infer a causal relationship, which is an inherent characteristic of all cross-sectional studies. Second, workplace SF policy was self-reported without verification, and the definitions of workplace SF policy of the respondents may vary. Third, the companies that joined the ABWMC programme were mainly located in developed areas of China, so our data are not a nationally representative sample. Fourth, the current study was limited to interested companies, which potentially introduced selection bias. Fifth, although we made our best attempt to remain anonymous, sometimes the anonymous is impossible. The survey was sent to employees by email or WeChat, so the IT department could match the questionnaire to each employee. The completed questionnaires were further reviewed by staff at Peking University, who then contacted the respondents for clarifications if any problems were detected. Sixth, we only measured SHS exposure in the workplace, and SHS exposure from elsewhere was neglected. In addition, we did not measure smoking reduction or the duration of workplace SF policies and policy enforcement, including incentives and penalties. Seventh, the survey questionnaire consists of researchers and organized companies, and some of the questions may not be consistent with standard questions in the Global Adult Tobacco Survey, such as SHS exposure. However, given the number of scholars applying this questionnaire to conduct tobacco-related research [15, 16, 25], the overall picture is believed to be meaningful.

Despite these limitations, our findings have practical policy implications. The results of the present study can encourage policymakers and health practitioners to accelerate the implementation of SF policy and contribute to a broader SF culture in the workplace. In particular, because workplace SF policy is weakly associated with stronger quitting intention, SF policies should be provided in combination with smoking cessation services. Regarding the results of the ABWMC programme, our findings will be disseminated to the participating companies, which may encourage those that struggle to catch up, establish more comprehensive workplace SF policies and improve the health status in China.

## Conclusion

Using ABWMC programme data, this study provides a comprehensive picture of the association of SF workplace policies with possible smoking-related behaviour using Chinese data. Although most companies have established workplace SF policies, the overall prevalence of SHS exposure remains very high. A workplace SF policy is associated with lower SHS exposure, fewer current smokers and higher awareness of smoking harm. These findings provide valuable evidence to promote such policies in all workplaces.

## Supplementary Information

Additional file 1: **Research Questionnaire**

## Data Availability

The datasets used and analysed during the current study available from the corresponding author on reasonable request.

## Abbreviations

SF: Smoke-free
SHS: Second-hand smoke
FCTC: Framework Convention on Tobacco Control
SHA: Smoking harm awareness
QI: Quitting intention
ABWMC: Asia Best Workplace Mainland China
