# Supplementary material for: The association of workplace smoke-free policies on individual smoking and quitting-related behaviours
Source: BMC Public Health. 2021 Dec 20;21:2308. doi: 10.1186/s12889-021-12395-z (PMC8686617; doi:10.1186/s12889-021-12395-z)
Supplement: Supplementary file 1 — Additional file 1: Research Questionnaire [file 12889_2021_12395_MOESM1_ESM.docx]

**Research Questionnaire**

**(Main part, translation version)**

**Demographic information**

1.Instituion： 2.Name：

3.Age： 4.Sex： □Male □Female

5. Ethnic：□Han □Others

6.Education：□middle school and lower □high school □college □master and above

7.Marrage： □single □married □divorced □widowhood

8. 2017 Yearly income：□<100000 yuan □100000-150000 yuan □150000-200000 yuan □ 200000-300000 yuan □ >300000 yuan

9. Do you have the following chronic diseases?？

□Hypertension□Hyperlipidemia □Diabetes □ Gastroenteritis □Heart disease □Lumbar or spinal diseases □Cancer□Hepatobiliary diseases □Anxiety □Others □No

**Health knowledge**

10. Do you know how much body mass index (BMI) is obese according to our reference standard?

□18 □24 □27 □30 □ I do not know

11. To the best of your knowledge, which diseases can be caused by smoking?

□stroke □heart disease □lung cancer □cardiovascular disease □COPD □asthma □ I don’t know.

12. To the best of your knowledge, which diseases can be caused by second head smoke?

□Adult heart disease □Lung disease in children □Adult lung cancer □ I don’t know.

13. How often do you search for health knowledge?

□Always □Very often □Sometimes, □Occasionally □Never.

**Health behavior**

14.How many days do you usually suffer from SHS exposure at workplace more than 15 minutes a week?

□ Almost every day, □ 4-6 days, □1-3 days or □Never’

15. What is your actual sleep time per night for the past month?

□less than 6 hours □6-8 hours □longer than 8 hours

16.Do you smoke now？

□Yes, every day □Yes, but only occasionally □I have quit □Never

17.How much cigarette you smoke every day? _____________

18.Are you going to quit smoking?

□ yes, within a month □ yes, within 6 months, □yes, but not within 6 months □no plan for quitting

19. Do you drink alcohol?

□Yes, every day □Yes, but only sometimes □Yes, only occasionally □Never

**Others**

20.Does your company provide you with tobacco related health education?

□ Yes □ No

21. Do you think your working environment is healthy?

□ I totally agree □I Agree □ Just ok, □ I do not agree □ I totally disagree

22. Do you think your company’s policy can protect your health?

□ I totally agree □I Agree □ Just ok, □ I do not agree □ I totally disagree

23.What is your position in this company?

□ Administrative position□ Not administrative position

24. In the past six months, how many days do you work overtime on average every week?

□ Everyday □4-6 days □ 1-3 days □ Never

25.Do you have night-shift duty

□Yes □No

26. Does your company have Smoke-Free policies?

□ No SF policies □ SF policies that permit smoking in parts of the indoor area

□SF policies that completely ban smoking inside the building □ I have no idea
